# Pharmacological effects of Chatuphalatika in hyperuricemia of gout

**DOI:** 10.1080/13880209.2017.1421235

**Published:** 2018-01-03

**Authors:** Vilasinee Hirunpanich Sato, Bunleu Sungthong, Prasob-Orn Rinthong, Narawat Nuamnaichati, Supachoke Mangmool, Savita Chewchida, Hitoshi Sato

**Affiliations:** a Department Pharmacology, Mahidol University, Bangkok, Thailand;; b Pharmaceutical Chemistry and Natural Products Research Unit, Mahasarakham University, Maha Sarakham, Thailand;; c Department Food Chemistry, Mahidol University, Bangkok, Thailand;; d Department of Pharmacokinetics and Pharmacodynamics, School of Pharmacy, Showa University, Tokyo, Japan

**Keywords:** Anti-inflammatory, xanthine oxidase, potassium oxonate-induced hyperuricemia, uric acid, RAW 264.7 cells

## Abstract

**Context:** Chatuphalatika (CTPT), is a Thai herbal formulation mixture of *Phyllanthus emblica* Linn. (Euphorbiaceae), *Terminalia belerica* Linn. (Combretaceae), *T. chebula* and the fruit of *T. arjuna* (Roxb.) Wight & Arn. CTPT is considered to exert anti-inflammatory and antihyperuricemic effects, but there have been no reports to demonstrate these pharmacological effects in a quantitative manner.

**Objectives:** To investigate the antioxidative, anti-inflammatory and antihyperuricemic effects of CTPT.

**Materials and methods:** Antioxidant activities of CTPT extracts were measured *in vitro* by DPPH, ABTS and FRAP assays, and anti-inflammatory effect by measuring inflammatory mediator production induced by lipopolysaccharide (LPS) in RAW264.7 macrophages. The mechanism of the hypouricemic effect was investigated using oxonate-induced hyperuricemic ddY mice treated with oral administrations of CTPT at 250, 500 and 1000 mg/kg.

**Results:** Antioxidant activities of CTPT measured by ABTS and FRAP assays were 1.35 g TEAC/g extract and 10.3 mmol/100 g extract, respectively. IC_50_ for the inhibition of DPPH radical was 13.8 µg/mL. CTPT (10 µg/mL) significantly downregulated the mRNA expression of TNF-α and iNOS in RAW 264.7 cells. Lineweaver–Burk analysis of the enzyme kinetics showed that CTPT inhibited xanthine oxidase (XOD) activity in a noncompetitive manner with the *K_i_* of 576.9 µg/mL. Oral administration of CTPT (1000 mg/kg) significantly suppressed uric acid production by inhibiting hepatic XOD activity, and decreased plasma uric acid levels in hyperuricemic mice by approximately 40% (*p* < 0.05).

**Conclusions:** This study demonstrated for the first time the antioxidative, anti-inflammatory and antihyperuricemic effects of CTPT *in vivo* and *in vitro*, suggesting a possibility of using CTPT for the treatment of hyperuricemia in gout.

## Introduction

Hyperuricemia has been established as a major risk factor in gout, which is caused by excess uric acid in blood due to increased production of uric acid and/or impaired renal urate excretion. The accumulation of urate, which results in the deposition of urate crystals in joints, causes inflammation and severe pain (Mancia et al. 2015). During purine metabolism, increased xanthine oxidase (XOD) activity leads to the production of superoxide and uric acid. Until now, it has been unclear whether uric acid is an independent factor leading to cardiovascular disease and metabolic abnormalities. However, hyperuricemia is often accompanied by hypertension, diabetes, dyslipidaemia, chronic renal disease and obesity. Moreover, several meta-analyses of observational studies have shown that hyperuricemia can significantly increase the risk of coronary heart disease (CHD) events (Li et al. 2016). Therefore, it is believed that the uric acid level merely serves as a marker of cardiovascular disease (Kelkar et al. 2011; Saralamp et al. 2011; Billiet et al. 2014). Epidemiological and biochemical studies examining uric acid formation have shown that uric acid leads to a worsening prognosis and increased cardiovascular events as do the free radicals and superoxides formed by xanthine oxidase activity. The combination of uric acid formation and free radicals ultimately leads to coronary endothelial dysfunction and worsening of myocardial oxidative stress (Billiet et al. 2014). Allopurinol is the first-line drug for the treatment of hyperuricemia; however, an inadequate response is observed in some patients. Moreover, this drug may cause severe allergic hypersensitivity, such as Stevens–Johnson syndrome, especially in patients carrying the HLA-B*5801 allele (Kelkar et al. 2011). Therefore, studies have focused on the development of agents possessing a safe combination of anti-inflammatory, antioxidant and antihyperuricemic activities.

Chatuphalatika (CTPT) is a herbal formulation which has been used in traditional Thai medicines (Saralamp et al. 2011). It consists of equal parts of four fruits, three of which have the same compositions as a well-known Ayurvedic formulation of *Phyllanthus emblica* Linn. [or *Emblica officinalis* Gaertn] (Euphorbiaceae), *Terminalia belerica* Linn. (Combretaceae), *T. Chebula,* and the fruit of *T. arjuna* (Roxb.) Wight & Arn. Formulations of CTPT have been used as a laxative, for colon cleansing, detoxification and calorie control (Saralamp et al. 2011). The medical usage of CTPT is considered to be similar to triphala, particularly in terms of its antioxidant activity.

CTPT has a composition similar to triphala, which has been shown to be beneficial for gouty arthritis (Naik et al. 2006; Sabina and Rasool 2008; Kalaiselvan and Rasool 2015). We have reported the pharmacological mechanisms associated with triphala (Sato et al. 2017). Recently, CTPT has been advertised as a food supplement which is beneficial to several diseases, including hyperuricemia in gout. However, mechanistic studies to support these beneficial pharmacological activities (i.e., antihyperuricemic and anti-inflammatory effects) of CTPT have been lacking so far. Therefore, the objectives of the present study were to evaluate the antioxidative and antihyperuricemic effects of CTPT under *in vitro* and *in vivo* conditions. In particular, direct *in vivo* evidence for the effect of CTPT on XOD activity was evaluated using potassium oxonate-induced hyperuricemic mice. An enzyme kinetic analysis using Lineweaver–Burk plots was performed to assess the type of XOD inhibition by CTPT. Moreover, the anti-inflammatory effects of CTPT were determined using lipopolysaccharide (LPS)-stimulated RAW 264.7 macrophages.

## Materials and methods

### Materials

Gallic acid, ellagic acid, Folin–Ciocalteu reagent and oxonic acid potassium salt were purchased from Sigma Aldrich Chemical Co. (St. Louis, MO). Potassium pyrophosphate, dimethyl sulfoxide (DMSO), acetonitrile, methanol, carboxy methyl cellulose sodium (CMC-Na) salt, uric acid, allopurinol and xanthine were obtained from Wako Pure Chemicals (Osaka, Japan). Ultra-purified water was produced from Milli-Q academic A10 with 0.22 µm Millipak^®^ filter (Millipore, Damstadt, Germany). Xanthine oxidase purified from butter milk was obtained from Oriental Yeast Co., Ltd (Tokyo, Japan). All other chemical reagents were of analytical grade and used without further purification.

### Plant material

The fresh fruits of *P. emblica*, *T. chebula*, *T. belerica* and *T. arjuna* were collected from Doi saket district, Chiang Mai, Thailand in July 2014. Plant materials were authenticated as previously described (Saralamp et al. 2011) by Assist. Prof. Wanida Caichampoo, Pharmaceutical Chemistry and Natural Product Research Unit, Faculty of Pharmacy, Mahasarakham University, Maha Sarakham, Thailand. The voucher specimens of *P. emblica* (MSU.PH-EUP-PE01), *T. chebula* (MSU.PH-COM-TC01), *T. belerica* (MSU.PH-COM-TB01) and *T. arjuna* (MSU.PH-COM-TA01) were deposited at Faculty of Pharmacy, Maha sarakham University, MahaSarakham, Thailand. The seed of each fruit was removed, and the seedless fruits were then dried in a hot air oven at 60 °C for 48 h. The dried seedless fruits were blended using a blender and sieved through mesh no.14.

### Preparation of CTPT extract

Water extract of CTPT was prepared in a dried powder form. During the extraction process for CTPT, 100 mL sterile mid-stream human urine and 900 mL distilled water were used as solvent. *P. emblica*, *T. chebula*, *T. belerica* and *T. arjuna* (15 g each) were thoroughly mixed and macerated in a closed container for 7 days. The liquid part was filtered through filter paper. The filtrate was converted to powder by freeze drying. The obtained extract was kept in a closed container protected from light at −20 °C until further analysis.

### Quantification of ellagic acid and gallic acid in CTPT extract by high-performance liquid chromatographic (HPLC) method

Ellagic and gallic acids were used as chemical markers for the CTPT extract. The contents of both compounds were determined using HPLC as previously described (Charoenchai et al. 2016). Stock solutions of standard ellagic acid (0.1 mg/mL) and gallic acid (1 mg/mL) in methanol were freshly prepared prior to use. Desired concentrations of ellagic and gallic acids were subsequently diluted in methanol using the stock solutions. CTPT extract was dissolved in methanol at 2 mg/mL, and then filtered through a 0.45-µm PTFE syringe filter (Raphile Bioscience, China). The filtrates (20 µL) were introduced into the HPLC system in triplicate.

HPLC quantitative analysis was performed on a Shimadzu SCL-10 A VP equipped with LC-10 AD binary pumps and a SPD-M20A photodiode array detector (Kyoto, Japan). Data analysis was carried out using Class-VP version 6.1. Separation was performed on an Eclipse XDB-C18 column (250 × 4.6 mm, 5 µm) with an Eclipse XDB-C18 guard column (12.5 × 4.6 mm, 5 µm) at an ambient temperature. The HPLC mobile phase consisted of 0.05% trifluoroacetic acid (A) in water and acetonitrile (B) with a flow rate of 1 mL/min. The gradient elution was programed at 0–1 min, 5% B; 1–4 min, 5–10% B; 4–12 min, 10–15% B; 12–32 min, 15–35% B; 32–35 min, 35–50% B; 35–37 min, 50–100% B; 37–40 min, 100% B; 40–41 min, 100–5% B; 41–45 min, 5% B. The sample (20 µL) was injected into the HPLC system, and the absorbance was recorded at 270 nm.

Under the chromatographic conditions employed, the contents of ellagic and gallic acids in the CPTP extract were calculated from a calibration curve with a linearity of *y* = 0.139*x* − 0.0568 (*r^2^* = 0.99) and *y* = 0.0255*x* − 0.2194 (*r^2^* = 0.99), respectively, in the detection ranges of 20–160 and 40–320 µg/mL, respectively.

### Antioxidative assays

2,2-Diphenyl-1-picrylhydrazyl hydrate (DPPH) radical scavenging assay was performed as previously described (Thaipong et al. 2006). CTPT or ascorbic acid (as a positive control) was dissolved in water to obtain the final concentrations of 10, 100, 200, 400, 800 and 1000 µg/mL. Each solution (20 µL) was added to a 96-well microtitre plate containing 180 µL of 0.1 mM DPPH in methanol. After a 30 min incubation in the dark at room temperature, the absorbance at 517 nm was measured using a microplate reader (Infinited^®^ M200, Tecan, Switzerland). The DPPH radical scavenging activity percentage was calculated using Equation (1) as follows:

(1) $$DPPH\;scavenging\;activity\;\left(\%\right)=\frac{\left({Absorbance}_{control}\;–\;{Absorbance}_{sample}\right)}{{Absorbance}_{control}}\times 100$$

The DPPH radical-scavenging activity (%) was plotted against the plant extract or ascorbic acid concentration (µg/mL) to determine the concentration to decrease DPPH radical-scavenging by 50% (called IC_50_).

### 2,2-Azinobis 3-ethyl-benzothiazoline-6-sulphonic acid (ABTS) assay

For ABTS assay, the procedure followed a previously described method (Thaipong et al. 2006) with some modifications. The stock solutions containing 7 mM ABTS and 2.45 mM potassium persulfate were prepared, and the working solution was prepared by mixing the two stock solutions in equal quantities for 12–16 h in the dark at room temperature. The solution was then diluted by mixing 1 mL of ABTS solution with 24 mL of methanol to obtain an absorbance of 1.100 ± 0.020 units at 734 nm using a microplate reader (Infinited^®^ M200, Tecan, Switzerland). Fresh ABTS ˙ ^+^ solution was freshly prepared for each assay. The sample (10 µL) was mixed with 200 µL of ABTS ˙ ^+^ radical cation solution in 96-well plates. The absorbance was determined at 734 nm using a microplate reader (Infinited^®^ M200, Tecan, Switzerland). All determinations were carried out in triplicate. Trolox was used as a standard. The results are expressed as the mg Trolox equivalent antioxidant capacity (TEAC)/g extract.

### Ferric reducing antioxidant power (FRAP) assay

For FRAP assay, the procedure was adapted from a previous study (Thaipong et al. 2006). Briefly, the sample (500 µL) was mixed with 500 µL of 0.2 M potassium phosphate buffer (pH 6.6) and 500 µL of 1% w/v potassium ferricyanide solution. The mixture was incubated at 50 °C for 20 min, and then trichloroacetic acid (2 mL) was added to stop the reaction. In a 96-well plate, the supernatant from the mixture (100 µL) was added to 100 µL of deionized water and 20 µL of 0.1% w/v ferric chloride solution. The procedure was carried out in triplicate and allowed to stand for 30 min before measuring the absorbance at 700 nm using a microplate reader (Infinited^®^ M200, Tecan, Switzerland). Ferrous sulphate was used as a standard. The absorbance is expressed as the mmol ferrous sulphate equivalent/100 g extract.

### Total phenolic content assay

Total phenolic contents in CTPT aqueous extract were measured using a rapid microplate Folin–Ciocalteu method (Attard 2013). CTPT extract was diluted by methanol to obtain a final concentration of 1 mg/mL (called as CPTP sample). Gallic acid was dissolved in methanol in the range of 50–790 ng/mL. Folin–Ciocalteu working solution was prepared by 10-fold dilution with deionized water, and its portion (100 µL) was mixed with 10 µL of CTPT sample or gallic acid solution (as a phenolic marker). After the addition of Na_2_CO_3_ solution (80 µL), the reaction mixture was allowed to stand for 20 min at room temperature, and the total phenolic content was determined by UV spectrophotometry at 630 nm. Total phenolic content in CTPT extract was thus quantitated and represented as gallic acid equivalent (GAE) per gram of extract.

### Determination of total flavonoid content

The total flavonoid content in CTPT extract was determined by an aluminium chloride complex forming assay as previously described (Thaipong et al. 2006). The sample (100 µL) was mixed with 100 µL of 2% (w/v) AlCl_3_ solution in methanol, and incubated in a 96-well plate for 10 min at room temperature. The absorbance was analyzed at *λ*_max_ = 415 nm using a microplate reader (Infinited^®^ M200, Tecan, Switzerland). The total flavonoid determination was performed in triplicate. Quercetin was used as a standard, and flavonoid content was determined as the quercetin equivalent (QE) mg per gram of CTPT extract.

### Anti-inflammatory effect on LPS-stimulated RAW 264.7 macrophages

#### Cell culture

RAW 264.7 cells were obtained from Assoc. Prof. Primchanien Moongkarndi, Faculty of Pharmacy, Mahidol University, Thailand. RAW 264.7 cells were cultured and maintained in Dulbecco’s Modified Eagle’s Medium (DMEM) supplemented with 10% foetal bovine serum (FBS) and 1% penicillin/streptomycin (P/S), and incubated in a humidified atmosphere of 5% CO_2_/95% air at 37 °C. After the RAW 264.7 cells were grown to 80% confluence, they were passaged by treatment with 0.25% trypsin-EDTA solution to maintain the cells in an exponential growth stage. The medium was changed to serum-free DMEM before experiments.

### Cell viability test

Cell viability was measured by a modified 3-(4,5-dimethylthiazol-2yl)-2,5-diphenyl-2 H-tetrazolium bromide (MTT) assay (Stockert et al. 2012). Briefly, RAW 264.7 cells (1 × 10^4^ cells/well) were cultured in 96-well plates at a starting density of 1 × 10^4^ cell/well and incubated overnight. The cells were divided into control and treatment groups. The control group was exposed to varied concentrations of a vehicle (H_2_O and DMSO), while the treatment groups were treated with various concentrations of CTPT (0.01–500 µg/mL diluted with H_2_O and DMSO) for 24 h. After incubation, the culture medium was replaced with MTT solution (100 µL; 1 mg/mL dissolved in DMEM), and the RAW 264.7 cells were incubated for an additional 4 h. The medium was then removed by aspiration, and 100 µL DMSO was added to dissolve the insoluble formazan product. The samples were mixed thoroughly, and the optical density (OD) of each well was measured at 570 nm with a microplate reader (Infinited^®^ M200, Tecan, Switzerland). The results were calculated as percentage of viable cells compared with control cells. All experiments were performed in parallel in triplicate and repeated four times.

### Measurement of the mRNA expression of TNF-α, IL-1β, IL-6, iNOS and COX-2

RAW 264.7 cells were seeded at a density of 1 × 10^6^ cells/well in DMEM supplemented with 10% FBS and 1% P/S and maintained in a humidified 37 °C, 5% CO_2_ incubator for 24 h. The culture medium was then replaced with DMEM supplemented with 1% FBS and 1% P/S. The RAW 264.7cells were divided into four groups as follows: (1) cells that were not stimulated with LPS; (2) cells that were stimulated with 1 µg/mL of LPS for 12 h; (3) cells that were pretreated with the dilution of CTPT (1 µg/mL) for 3–4 h before being stimulated with 1 µg/mL of LPS for 12 h; 4) cells that were pretreated with 0.1 µM dexamethasone (positive control) for 3–4 h before stimulation with 1 µg/mL of LPS for 12 h (Guimarães et al. 2013).

The total RNA from RAW 264.7 cells was extracted using the GeneJET RNA Purification Kit (Thermal Fisher Scientific, Waltham, MA). The expression levels of cyclooxygenase-2 (*COX-2*), inducible nitric oxide synthase (*iNOS*), transforming growth factor-β (*TGF-β*) and tumor necrosis factor-α (TNF-α) were determined by quantitative reverse transcriptase polymerase chain reaction (RT-qPCR) using KAPA SYBR FAST One step RT-qPCR kits (KAPA Biosystems, Wilmington, MA) according to the manufacturer’s protocol. The gene-specific primers for RT-qPCR (mouse) were designed as shown in Table 1. RT-qPCR was performed under the following conditions: reverse transcription at 42 °C for 5 min; reverse transcriptase (RT) inactivation and DNA polymerase activation at 95 °C for 2–5 min; combined annealing, extension and data acquisition at 95 °C for 3 s and at 55 °C for 30 s (40 cycles); and a final extension at 72 °C for 1 min followed by 25 °C for 2 min. The expression of targeted genes was normalized to GAPDH and expressed as the fold change over the non-treated group.

**Table 1.** The gene specific primers for RT-qPCR (mouse).

Gene specific primer		Sequences
GAPDH	Sense	5′-GCCTGCTTCACCACCTTC-3′
	Antisense	5′-GGCTCTCCAGAACATCATCC-3′
COX-2	Sense	5′-TGCATGTGGCTGTGGATGTCATCAA-3′
	Antisense	5′-CACTAAGACAGACCCGTCATCTCCA-3′
iNOS	Sense	5′-GTGTTCCACCAGGAGATGTTG-3′
	Antisense	5′-CTCCTGCCCACTGAGTTCGTC-3′
TGF-β1	Sense	5′-TGGAGCAACATGTGGAACTC-3′
	Antisense	5′-TGCCGTACAACTCCAGTGAC-3′
TNF-α	Sense	5′-TACTGAACTTCGGGGTGATTGGTCC-3′
	Antisense	5′-CAGCCTTGTCCCTTGAAGAGAACC-3′

### Effect of CTPT extract on *in vitro* xanthine oxidase (XOD) activity

The effect of the aqueous extract of CTPT on XOD activity was assayed *in vitro* according to a previous study with some modifications (Kong et al. 2000). CTPT extract was initially dissolved in DMSO and diluted with distilled water (final concentration of DMSO less than 0.2% in the reaction mixture). Then, 20 µL of XOD enzyme (0.08 U) dissolved in 0.1 M pyrophosphate (pH 7.4) was freshly prepared and gently mixed with 1 mL of CTPT extract (30–1000 µg/mL) or buffer (as a negative control). Allopurinol (0.03–0.5 µM) was used as a positive control. Subsequently, the reaction mixtures were incubated for 10 min at 25 °C. The enzyme reaction was initiated by the addition of 2 mL of 120 µM xanthine (as a substrate) dissolved in 0.1 M pyrophosphate buffer (pH 7.4) and incubation at 25 °C for 10 min. The reaction was then terminated by addition of 1 mL of 1 N HCl, and the concentrations of uric acid were determined by HPLC, as described later.

The percentage inhibition of the XOD-mediated uric acid formation by CTPT extract or allopurinol was calculated using Equation (2) as follows:

(2) $$Inhibitory\;activity\;of\;XOD\;\left(\%\right)=\left(1-\frac{{UA}_{sample}}{{UA}_{control}}\right)\times 100$$

where UA_control_ is the concentration of uric acid in the negative control, and UA_sample_ is the concentration of uric acid in the sample.

Then, the IC_50_, defined as the concentration required inhibiting XOD activity by 50%, was calculated by a linear regression analysis of the dose-response curve between the percentage of inhibitory activity and log concentration.

### Enzyme kinetic analysis of CTPT extract on XOD inhibition *in vitro*

Determination of type of XOD inhibition by CTPT extract was performed by Lineweaver–Burk plot analysis. Three concentrations of CTPT extract (125, 250 and 500 µg/mL) assessed in different concentrations of xanthine as a substrate (15, 30, 60 and 120 µM). The enzyme reaction was performed as described above. The inhibitory constant (*K*_i_) for XOD inhibition by CTPT extract was determined by a non-least squares regression of the observed data following the equation (Equation 3) using Solver Add-in equipped with Microsoft Excel 2010:

(3) $$v=\frac{\;V_{\max}\cdot \;S}{K_{\text{m}}+S\left(1+\frac{I}{K\text{i}}\right)}$$

where *v* and *V*_max_ represent the initial and maximum velocities of the uric acid formation, respectively (mmol/min), *K*_m_ represents the Michaelis constant (µM), and *S* and *I* represent the substrate (µM) and inhibitor concentrations (µg/mL), respectively. For the non-linear optimization, the generalized reduced gradient (GRG) algorithm of the Solver add-in implemented in Microsoft Excel 2013 was employed.

### *In vitro* effects of CTPT extract on plasma uric acid levels in potassium oxonate induced-hyperuricemic mice

Male ddY mice (30–40 g, 6 weeks old) were housed at the animal centre of Showa University, Tokyo, Japan, at a constant temperature (25 ± 1 °C) with a 12 h light–dark cycle and free access to standard diet and water *ad libitum*. Mice were acclimatized for 7 days prior to starting the experiment. The animal care and experimental protocol were approved by the animal ethics committee of Showa University (permission number 27061).

The mice were randomly divided into six groups (5–6 mice per group). Group 1 contains the normal control mice that were pretreated orally with water and had no further treatment. Hyperuricemic mice were developed according to a previous study (Smith et al. 1979) with modifications. Groups 2 to 6 were induced to be hyperuricemic by intraperitoneal (*i.p*.) injection of 300 mg/kg potassium oxonate (as an uricase inhibitor) dissolved in normal saline at 8:00 AM, and after 10 min each group was orally administered the following: water; group 3: allopurinol (5 mg/kg); groups 4–6: CTPT extracts (250, 500 and 1000 mg/kg), respectively. Each treatment was given consecutively for 7 days. On the seventh day at 1 h after treatment (9:00 AM), mice in groups 2 to 6 were orally administered at 300 mg/kg xanthine (as an XOD substrate) dispersed in 0.5% CMC-Na, while group 1 was orally administered at 0.5% CMC-Na. At 2 h after the pretreatment (approximately 11:00 AM), the mice were sacrificed by anaesthesia using thiopental (40 mg/kg, i.p.), and whole blood was collected *via* cardiac heart puncture into a tube containing heparin. The blood samples were centrifuged for 5 min at 3000 *g*, 4 °C, and plasma samples were obtained and stored at −20 °C until analysis. Subsequently, mouse livers and kidneys were rapidly excised, and washed 2–3 times with cold 0.9% normal saline for further experiments to determine residual XOD activities in the livers *ex vivo* and to perform a western blot analysis, as described later.

### Effects of CTPT extract on residual activity of XOD in liver homogenates *ex vivo*

Residual activities of XOD in the excised livers were determined by measuring uric acid concentrations generated from xanthine. Briefly, the livers were homogenized in five volumes of cold 80 mM sodium pyrophosphate buffer, pH 7.4, and centrifuged at 3000 *g* for 10 min at 4 °C. After the lipid layer was removed, the remaining part was further centrifuged at 10,000 *g* for 60 min at 4 °C, and the obtained supernatants were used to measure the residual XOD activity.

A portion (100 µL) of each liver homogenate and 800 µL of 80 mM sodium pyrophosphate buffer (pH 7.4) were mixed and incubated at 25 °C for 10 min. Then, 500 µL of 120 µM xanthine solution was added, mixed and incubated for 30 min. The reaction was terminated after 0 and 30 min by adding 100 µL of 1 N HCl. Thereafter, the collected samples were centrifuged at 3000 *g* for 10 min, and uric acid concentrations were measured by HPLC. The total protein concentration in the liver homogenate was determined using Protein Assay Bradford Reagent (Wako Pure Chemicals, Osaka, Japan). The residual XOD activity was determined and expressed as nmole of uric acid formed/min/mg protein.

### Western blotting analysis of GLUT9 in the kidney cortex tissues

Kidney cortex tissues were collected and homogenized in lysis buffer containing 20 mM Tris HCl, pH 7.4, 2 mM EDTA, 1% Nonidet P-40, 10 mM NaF, 1 mM Na_3_VO_4_, 20% glycerol, 100 µM phenylmethylsulfonyl fluoride (PMSF), 5 µg/mL aprotinin and 5 µg/mL leupeptin. After centrifugation, the protein concentration in the kidney homogenates was determined using a Bio-Rad protein assay kit with bovine serum albumin as the standard. Samples were mixed with loading buffer and denatured by heating at 95 °C, 5 min prior to separation by SDS-PAGE. Separated proteins were transferred to a polyvinylidene fluoride (PVDF) membrane (Bio-Rad, Hercules, CA). After blocking nonspecific binding sites for 1 h with 5% skim milk, the membranes were individually incubated overnight with anti-GLUT9 (1:1000 dilution) as well as anti-GAPDH (1:2000 dilution) antibodies. Immunoblots were visualized with HRP-conjugated secondary antibodies and a chemiluminescence detection system (GE Healthcare, Piscataway, NJ).

### Determination of uric acid concentrations by HPLC

Plasma uric acid concentrations and residual XOD activities of liver homogenates were determined by reversed-phase HPLC with a TSkgel ODS-80Ts column (150 × 4.6 mm, 5 µm; Tosoh bioscience, Tokyo, Japan) on a HPLC system (Shimadzu Corp., Kyoto, Japan) consisting of a quaternary pump LC (LC-20AT) with a degasser (DGU-20A3), UV-Vis detector (SPD-20 A), and communication module (CBM-20 A); the analytical column was constantly maintained at 25 °C. The analytical method was modified from a previous study (Carro et al. 2010). Briefly, the aliquoted sample volume (100 µL) was thoroughly mixed with acetonitrile (100 µL), centrifuged at 3000 *g* for 5 min at 4 °C, and the supernatant was then filtered through 0.45 µM PTFE syringe filter (Raphile Bioscience, China). The filtrates (20 µL) were introduced into the HPLC system in triplicate. The HPLC isocratic mobile phase consisted of acetonitrile and 1 mM octane sulphonic acid, sodium salt in 7 mM K_2_HPO_4_, pH 3.0 (2.5:97.5) with a 1.0 mL/min flow rate. The amount of uric acid in the samples was determined at 292 nm and calculated using a calibration curve of spiked uric acid in blank plasma or buffer ranging from 6.25–100 µg/mL, after subtracting the level of endogenous uric acid.

Determination of uric acid in the *in vitro* experiments of XOD activity was also performed using HPLC as described previously. After terminating the reaction, the samples were directly filtered through a 0.45 µM PTFE syringe filter and then injected into the HPLC system. The amount of uric acid was quantified according to the calibration curve of standard uric acid solution from 1.56–50 µg/mL.

## Results

### Plant extraction and HPLC quantification of ellagic and gallic acids in CTPT extract

**Figure 1.**
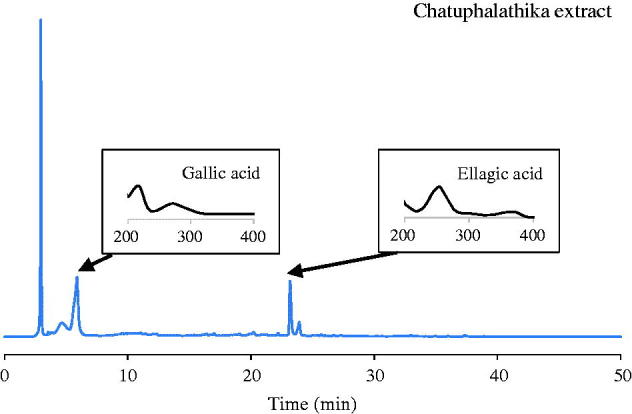
HPLC quantification of ellagic acid and gallic acid in CTPT extract.

CTPT extract was obtained as a dry powder with a brownish colour, and the percentage of yielded extract was 18.28%. A typical HPLC chromatogram of CTPT is shown in Figure 1. The corresponding peaks of gallic and ellagic acids in the extract were confirmed by the retention times and spectra compared with standard solutions. The amounts of ellagic and gallic acids were quantified as phytochemical markers to be 16.27 ± 0.60 and 172.50 ± 11.40 mg/g extract, respectively.

### Antioxidative effects

Total phenolic and total flavonoid contents were determined to be 423.03 ± 4.57 mg GAE/g extract and 6.31 ± 0.84 mg QE/g extract, respectively. The IC_50_ value for the inhibition of DPPH radical by CTPT was 13.78 ± 1.04 µg/mL, while that of ascorbic acid was 1.78 ± 0.31 µg/mL. Antioxidant activities measured by ABTS and FRAP were 1.35 ± 0.01 g TEAC/g extract and 10.27 ± 0.62 mmol/100 g extract, respectively.

### The effects of CTPT on inhibition of mRNA expression of LPS-induced inflammatory mediators in Raw264.7 macrophages

**Figure 2.**
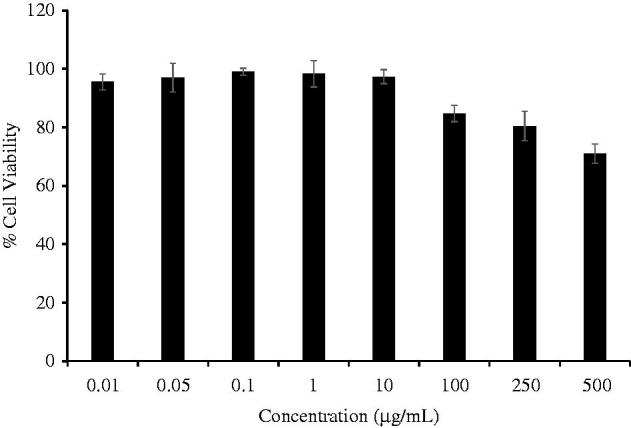
Effects of CTPT extract on the viability of RAW 264.7 cells. RAW264.7 cells were treated with various concentrations of CTPT extract (0.01–500 µg/mL) for 24 h. Cell viability was quantified, expressed as a percentage of cell viability, and shown as the mean ± SEM of four independent experiments.

**Figure 3.**
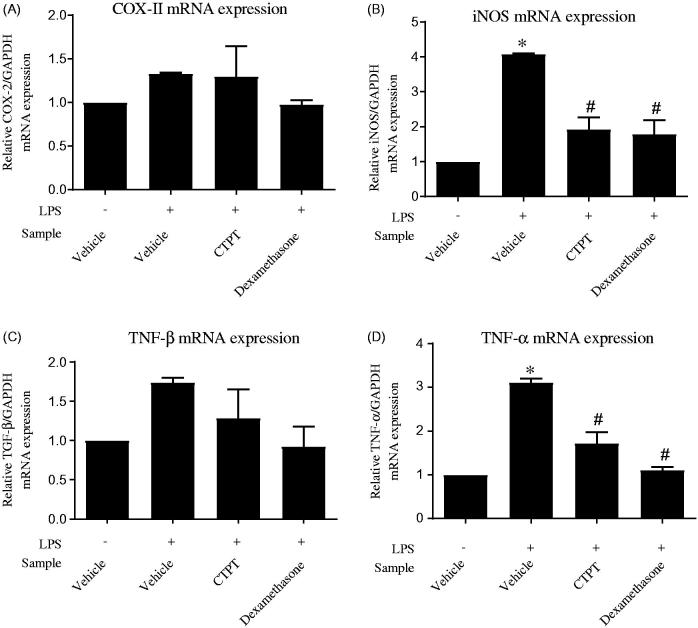
The effects of CTPT extract on inhibition of LPS-induced mRNA expression of inflammatory mediators in RAW264.7 cells. Cells were pretreated without or with CTPT (10 µg/mL) or 0.1 µM dexamethasone for 3 h and then stimulated with 1 µg/mL LPS for 12 h at 37 °C. Relative mRNA levels were quantified and are shown as the mean ± SEM of four independent experiments. *p < 0.05 compared with control; #*p* < 0.05 compared with LPS.

Figure 2 shows the viability of RAW 264.7 cells in the presence of CTPT extract. The data indicate that CTPT extract was not cytotoxic to RAW 264.7 cells at the concentration range of 0–500 µg/mL. Based on these results, 0.01–10 µg/mL concentrations of CTPT were selected for subsequent experiments, as their cytotoxicities were not obviously different compared with the control. CTPT was found to inhibit the increased mRNA expression levels of *COX-II* and *TGF-β* stimulated by LPS, as shown in Figures 3(A,C), respectively. Especially, treatment with CTPT at 10 µg/mL significantly inhibited the LPS-stimulated iNOS and TNF-α mRNA expression, as presented in Figures 3(B,D), respectively (*p* < 0.05).

### Effects of CTPT extract on *in vitro* xanthine oxidase activity

**Figure 4.**
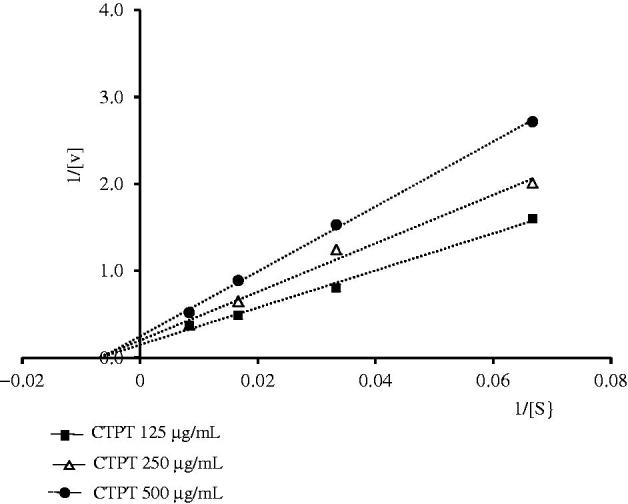
Lineweaver–Burk plots for the kinetic analysis of xanthine oxidase activity inhibited by CTPT extract. The (▪), (Δ), (•) represent CTPT extract at concentrations of 125, 250 and 500 µg/mL, respectively.

CTPT extract inhibited the XOD activity *in vitro* in a concentration-dependent manner and the IC_50_ of CTPT extract was 390.7 ± 7.1 µg/mL, while that of the positive control, allopurinol, was 3.78 ± 0.08 µg/mL. Lineweaver–Burk analysis of the *in vitro* enzyme kinetic data (Figure 4) showed that the presence of CTPT caused a decrease in the *V*_max_ of XOD compared with the control with very little change in the *K*_m_ of xanthine, indicating a typical reversible, noncompetitive inhibition of enzymatic reaction. The *K*_i_ value of the CTPT extract was 576.9 µg/mL.

### *In vivo* effects of CTPT extract on plasma uric acid levels in potassium oxonate-induced hyperuricemic mice

**Figure 5.**
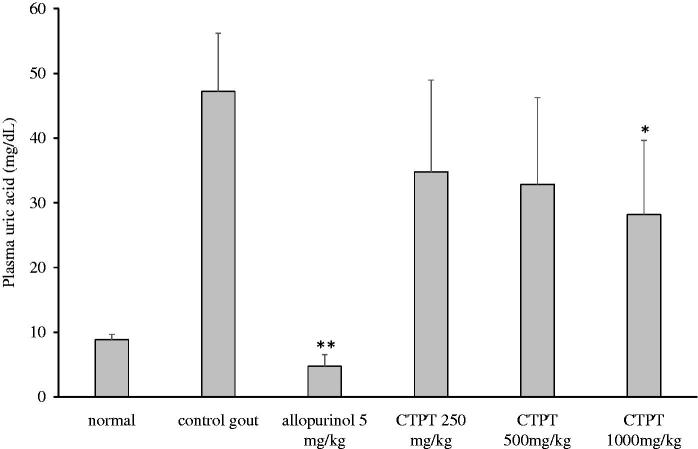
Effect of CTPT extract on the plasma uric acid concentration in potassium oxonate-induced hyperuricemic mice. Group 1 represents the normal control group; group 2 is untreated potassium oxonate-induced hyperuricemic mice; group 3 is potassium oxonate-induced hyperuricemic mice treated with allopurinol (5 mg/kg); groups 4, 5 and 6 are potassium oxonate-induced hyperuricemic mice treated with 250, 500 and 1000 mg/kg CTPT extract, respectively. Data are expressed as the mean ± SEM (*n* = 6). * and ** indicate statistically significant differences at *p* < 0.05 and *p* < 0.01, respectively, when compared to group 2.

*In vivo* effects of CTPT extract on uric acid concentration in plasma are presented in Figure 5. The uric acid level in the plasma of the control hyperuricemic group was elevated significantly after an *i.p.* injection of potassium oxonate compared with the normal control group (47.2 ± 8.5 *vs.* 8.8 ± 0.8 µg/mL, *p* < 0.01). Plasma uric acid concentration was significantly reduced by approximately 88% after pretreatment with allopurinol (group 3) compared with the non-treatment hyperuricemic group (*p* < 0.01). Treatment with oral doses of CTPT extracts (250 and 500 mg/kg) decreased uric acid concentrations by approximately 26% and 30%, respectively, but these values were not significantly different from that of the untreated hyperuricemic group. In contrast, treatment with 1000 mg/kg of CTPT significantly decreased plasma uric acid concentrations by approximately 40%, compared with the untreated hyperuricemic control group (*p* < 0.05).

### Effects of CTPT extract on residual XOD activity in liver homogenates *ex vivo*

Treatments with allopurinol (5 mg/kg) and CTPT extract (1000 mg/kg) markedly inhibited the formation of uric acid in the liver homogenates *ex vivo* through inhibition of XOD activity by 90.3% and 76.0%, respectively, compared with the untreated hyperuricemic group (*p* < 0.01 and *p* < 0.05, respectively) (Table 2). Treatment with CTPT extract (250 and 500 mg/kg) decreased the formation of uric acid by inhibiting XOD activity in the liver homogenates by approximately 30% and 43%, but these values were not significantly different from that of the untreated hyperuricemic group.

**Table 2.** Residual xanthine oxidase activity (XOD) in liver extracted from mice treated with chatuphalatika extract.

Groups	XOD activity (nanomole/min/mg protein)	Inhibition (%)
1	0.98 ± 0.07	–
2	1.84 ± 0.15	–
3	0.03 ± 0.001**	98.4
4	1.24 ± 0.29	32.6
5	1.10 ± 0.20	40.2
6	0.44 ± 0.12*	76.1

Data are the mean ± SEM, obtained from six mice per group.

* *p* < 0.05 compared with the untreated hyperuricemic group.

** *p* < 0.01 compared with the untreated hyperuricemic group.

Group 1 represents the normal control group; group 2 contains untreated potassium oxonate-induced hyperuricemic mice; groups 3 contains potassium oxonate-induced hyperuricemic mice treated with allopurinol (5 mg/kg); groups 4, 5 and 6 represent potassium oxonate-induced hyperuricemic mice treated with 250, 500 and 1000 mg/kg chatuphalatika extract, respectively.

### GLUT9 protein expression in kidney cortex tissues

**Figure 6.**
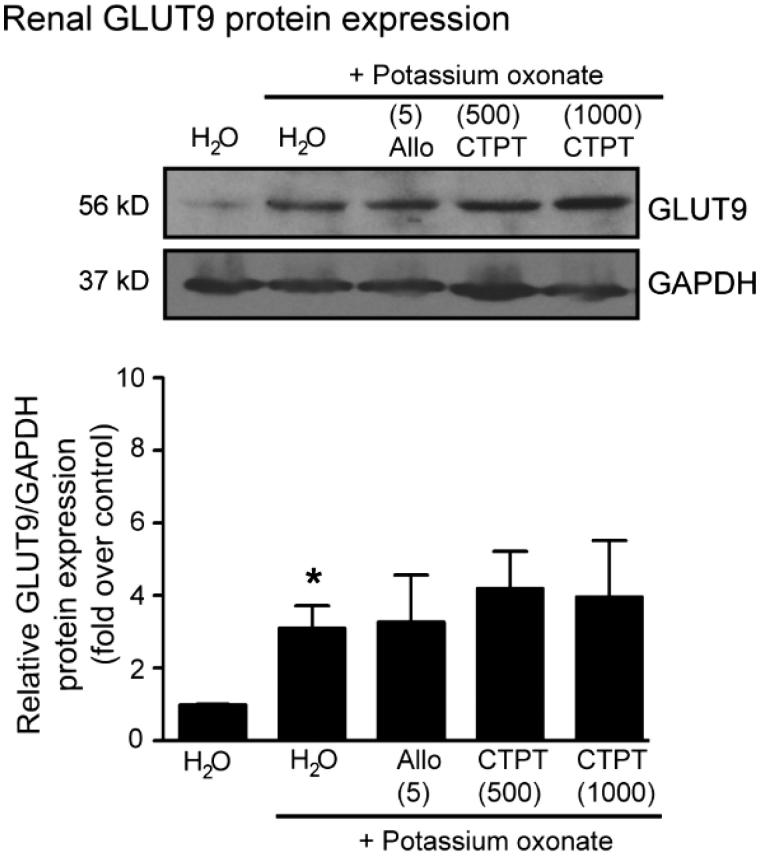
Effects of CTPT extract and allopurinol on GLUT9 protein expression in renal tissues in potassium oxonate-induced hyperuricemic mice. Normal control and untreated potassium oxonate-induced hyperuricemic mice groups were treated with water. Treatment groups were potassium oxonate-induced hyperuricemic mice treated with allopurinol (5 mg/kg) and 500 and 1000 mg/kg CTPT extract, respectively. At the end of the study, renal homogenates of mice were immunoblotted with anti-GLUT9 and anti-GAPDH antibodies. The relative GLUT9 protein levels were quantified and expressed as the fold increase over normal control mice and are shown as the mean ± SEM (*n* = 4) for each group. *p < 0.05 compared with control mice. CTPT and Allo represent chatuphalatika extract and allopurinol, respectively.

The results of Western blot analysis for GLUT9 in the kidney cortex tissues of potassium oxonate-induced hyperuricemia mice are shown in Figure 6. Significant increases in the renal GLUT9 protein levels were observed in hyperuricemia mice compared with normal mice. However, treatment with either allopurinol (5 mg/kg) or CTPT (500 and 1000 mg/kg) did not affect the GLUT9 protein expression.

### Determination of uric acid concentrations by HPLC

HPLC was used to determine the uric acid concentrations in samples obtained from *in vitro* and *in vivo* experiments. Uric acid in plasma and residual XOD activity in liver were quantified using a calibration curve of plasma uric acid. The calibration curve exhibited a good correlation (*r^2^* = 0.998) with a linear equation: *y* = 19,381*x* + 11,564. The analytical method also showed good precision of 0.90–7.60% over the studied concentration range. The percentage recovery of the uric acid extraction from plasma was 67.81 ± 3.64% (*n* = 3). The concentrations of plasma uric acid observed in all groups fell within the studied range. In the case of *in vitro* XOD activity, the calibration curve of standard uric acid solution showed a good linearity (*r^2^* = 0.9998) with a linear equation: *y* = 56781*x* + 18585. The precision of the analytical method ranged 0.61–1.57%.

## Discussion

The Thai herbal formulary, CTPT, consists of four herbs, *E. officinalis*, *T. belerica*, *T. chebula,* and the fruit of *T. arjuna,* in equal amounts. Various pharmacological effects in these plants have been reported, i.e., anticancer, antihyperglycaemic, antioxidant and antibacterial activities (Shinde et al. 2009; Biswas et al. 2011, 2012; Mandal et al. 2013). Several active phytochemical compounds are found at high concentrations in each plant, including phytosterol, lactones, flavonoids, phenolic compounds, tannins and glycosides (Saralamp et al. 2011). Several compounds are observed in CTPT extract, such as ellagic acid, gallic acid, chebulinic acid and chebulagic acid. Moreover, numerous agents derived from the plants have anti-gout potential. Phenolic compounds have been reported to inhibit uric acid production and uricosuric and anti-inflammatory effects (Ling and Bochu 2014). A previous report suggested that gallic acid and its derivative competitively inhibit XOD activity and suppress superoxide anion generation catalyzed by XOD (Masuoka et al. 2006). Ellagic acid exhibits an inhibitory effect on XOD activity in rats with methotrexate-induced intestinal damage (El-Boghdady 2011). As presented in Figure 1, ellagic and gallic acids were shown to be the major constituents in CTPT extract. Therefore, we employed these two compounds as standardized, bioactive markers of the extract, with a notion that they might contribute to the antioxidative, anti-inflammatory and antihyperuricemic effects of CTPT extract.

In human, uric acid is the terminal step of purine metabolism catalyzed by XOD, which also produces reactive oxygen species (ROS) such as superoxide. The major sources of XOD are the liver and small intestine, but there are local production sites of XOD at endothelium and myocardium, where ROS is also generated. Several studies found that XOD is up-regulated in many cardiovascular diseases, such as myocardial ischaemia and heart failure associated with enhanced oxidative stress (Kelkar et al. 2011; Billiet et al. 2014). There is clinical evidence that the increased uric acid level is associated with coronary disease, stroke and atherosclerosis (Hozawa et al. 2006). Therefore, counteracting the ROS generation is a recent strategy for cardiovascular diseases, together with controlling the uric acid concentration in blood (Billiet et al. 2014). Antioxidative effects of *P. emblica*, *T. belerica*, *T. chebula* and *T. arjuna* have been reported (Shinde et al. 2009; Biswas et al. 2011, 2012; Mandal et al. 2013) which may be the reason for strong antioxidant effects of CTPT that have been observed.

Gout is an inflammatory condition that is associated with the deposition of urate crystals, leading to pain and inflammation in the synovial joints (Mancia et al. 2015). Treatment of inflammation under hyperuricemia control is the major therapeutic approach. During inflammatory processes, several cytokines are released, i.e., TNF-α, TNF-β, iNOS and COX-2. Therefore, inhibition of these cytokines could be a promising anti-inflammation strategy. The anti-inflammatory effects of the combination of *P. emblica*, *T. belerica* and *T. chebula* in equal proportions, known as triphala, have been reported to inhibit expression of cytokines such as TNF-α, IL-1β, VEGF, MCP-1 and PGE2 in *in vivo* experimental gouty arthritis in rats (Kalaiselvan and Rasool 2015). Consistently, in this study, CTPT extract exhibited anti-inflammatory effects by significantly reducing the mRNA expression of inflammatory mediators, e.g., TNF-α and iNOS, in RAW264.7 cells. These findings should provide a scientific basis for investigations of the inflammatory effects of CTPT formulations in future clinical studies.

The present study is the first to provide direct evidence of the antihyperuricemic effect of CTPT extract in potassium oxonate-induced hyperuricemic mice. We found that an oral administration of CTPT extract (1000 mg/kg) prevented the increase in plasma uric acid levels in hyperuricemic mice. Moreover, the results obtained in the present *ex vivo* study showed that CTPT significantly decreased the formation of uric acid by inhibiting XOD activity in the liver, indicating that CTPT can be absorbed orally, distributed into the liver and thereafter inhibit the XOD activity. Unlike allopurinol, a strong competitive inhibitor of xanthine oxidase (Kelkar et al. 2011), CTPT is indicated to be a noncompetitive inhibitor, which preferably binds to another substrate binding site and subsequently inhibits the enzyme activity. Considering the *in vitro* and *in vivo* data together, it is suggested that CTPT has an antihyperuricemic effect in potassium oxonate-induced hyperuricemic mice due to its inhibitory action on XOD, which is mainly due to liver XOD inhibition after oral administration.

We have examined the antihyperuricemic effect of triphala in a previous study (Sato et al. 2017), and found that the 1000 mg/kg oral dose of triphala reduced plasma uric acid concentration by about 40% and exhibited about 80% reduction of xanthine oxidase activity in the liver, in potassium oxonate-induced hyperuricemic mice as compared with control, similarly with CPTP. Therefore, it was suggested that triphala and CTPT have comparable hypouricemic effects. However, as inspected from the present and previous studies, CTPT which contains *T. arjuna* in addition to triphala exhibits a higher level of total phenolic contents than that of triphala (423.03 ± 4.57 mg vs. 317.6 ± 9.2 GAE/g), resulting in a slightly stronger antioxidant effect with the IC_50_ values for inhibition of DPPH radicals being 13.78 ± 1.04 mg/mL vs. 21.97 ± 2.5 mg/mL for CTPT and triphala, respectively.

Hyperuricemia can be caused by either excessive production and/or low excretion of uric acid. Several transporters, such as URAT1, OAT4, OAT1/3 and GLUT9, are thought to be the essential for regulating uric acid reabsorption (So and Thorens 2010). GLUT9 is encoded by SLC2A9 and found in the lateral brush border membranes of the renal tubular epithelial cells, which mediates the reabsorption of uric acid into the blood. Inhibition of this transporter is one of the targets of uricosuric agents to treat hyperuricemia. In the present study, renal GLUT9 expression was up-regulated in potassium oxonate-induced hyperuricemic mice, indicating an increase in the reabsorption of uric acid in the renal tubules and thus resulting in elevated plasma urea. The present results showed that allopurinol, a strong hepatic XOD inhibitor, had no effect on renal GLUT9 expression in hyperuricemic mice, which is consistent with a previous study (Hou et al. 2012). Treatment with CTPT did not affect the expression of GLUT9 protein, suggesting that CTPT has no effect on urate GLUT9 transporter localized in the kidney. However, the effects of CTPT extract on other transporters, i.e., URAT1, OAT4 and OAT1/3 should be further investigated in future.

A toxicity test of CTPT has not yet been reported in the literature. Acute toxicity in rats is currently being investigated in our laboratory in accordance with the Organization for Economic Co-operation and Development (OECD) test guideline for acute oral toxicity (OECD 2001). The CTPT doses of 2000 and 5000 mg/kg body weight were administered to male and female Wistar rats for 14 days, and the body weight, food/water consumption, haematological values and mortality were compared with control rats at elapsed times. After 14 days, all these observed data were equivalent between CTPT-treated and control rats, indicating no sign of toxicity related to CTPT (unpublished data). Regarding information about the toxicity of each herb component, the extract of *T. chebula*, *T. bellerica* and *P. emblica* has been considered to be safe when orally administered daily for 10 days at doses up to 23.04 g/kg/day in rats (Chavalittumrong et al. 1996). The alcoholic fruit extract of *T. arjuna* (2000 mg/kg) did not produce any signs of toxicity or mortality.

Moreover, the human equivalent dose (HED) calculation based on body surface area was determined using Equation (4) as follows (Nair and Jacob 2016):

(4) $$\text{HED}\left(\frac{\text{mg}}{\text{kg}}\right)=\text{Animal}\;\text{dose}\left(\frac{\text{mg}}{\text{kg}}\right)\;\times \;\left(\text{Animal}\;K_{m}\right)/\left(\text{Human}\;K_{m}\right)$$

where the animal dose is the effective dose used in the animal, which was found to be 1000 mg/kg in this study. The animal *K_m_* and human *K_m_* are the correction factors estimated by dividing the average body weight (kg) of the species by its body surface area (m^2^), which are calculated to be 3, 6 and 37 for mouse, rat and human, respectively.

Therefore, the HED calculated from the effective CTPT dose in mice (1000 mg/kg) can be calculated to be 81 mg/kg. This dose is considered to be lower than that of the estimated toxic dose (more than 5000 mg/kg in rats, corresponding to HED of 811 mg/kg). However, chronic toxicities of CTPT should be further evaluated before considering clinical application of the herb formulation.

## Conclusions

This study demonstrated for the first time the antioxidative, anti-inflammatory and antihyperuricemic effects of CTPT extract *in vivo* and *in vitro.* In particular, *in vivo* antihyperuricemic effect of CTPT extract was explained by a mechanism of XOD inhibition in the liver, which was identified to be a noncompetitive type of inhibition. Moreover, CTPT extract exhibited anti-inflammatory effects by significantly reducing the mRNA expression of inflammatory mediators, e.g., TNF-α and iNOS, in RAW264.7 cells. Since the antioxidative and anti-inflammatory effects of CTPT are beneficial for the treatment of gout, the results obtained in this study suggest that CTPT can be used as a natural remedy for the treatment of hyperuricemia in gout.
